# Internal validation and comparison of predictive models to determine success rate of infertility treatments: a retrospective study of 2485 cycles

**DOI:** 10.1038/s41598-022-10902-9

**Published:** 2022-05-04

**Authors:** Ameneh Mehrjerd, Hassan Rezaei, Saeid Eslami, Mariam Begum Ratna, Nayyere Khadem Ghaebi

**Affiliations:** 1 Department of Computer Science, Faculty of Mathematics, Statistics and Computer Science, University of Sistan and Baluchestan, Zahedan, Iran; 2 Department of Medical Informatics, Amsterdam UMC, Location AMC, University of Amsterdam, Amsterdam, The Netherlands; 3 Department of Medical Informatics, Faculty of Medicine, Mashhad University of Medical Sciences, Mashhad, Iran; 4 Pharmaceutical Research Center, Mashhad University of Medical Science, Mashhad, Iran; 5 Department of Epidemiology and Public Health, School of Medicine, University of Nottingham, Nottingham, UK; 6 Department of Obstetrics and Gynecology, School of Medicine, Mashhad University of Medical Sciences, Mashhad, Iran

**Keywords:** Diagnosis, Experimental models of disease

## Abstract

Infertility is a significant health problem and assisted reproductive technologies to treat infertility. Despite all efforts, the success rate of these methods is still low. Also, each of these methods has side effects and costs. Therefore, accurate prediction of treatment success rate is a clinical challenge. This retrospective study aimed to internally validate and compare various machine learning models for predicting the clinical pregnancy rate (CPR) of infertility treatment. For this purpose, data from 1931 patients consisting of in vitro fertilization (IVF) or intra cytoplasmic sperm injection (ICSI) (733) and intra uterine insemination (IUI) (1196) treatments were included. Also, no egg or sperm donation data were used. The performance of machine learning algorithms to predict clinical pregnancy were expressed in terms of accuracy, recall, F-score, positive predictive value (PPV), brier score (BS), Matthew correlation coefficient (MCC), and receiver operating characteristic. The significance of the features with CPR and AUCs was evaluated by Student's *t* test and DeLong’s algorithm. Random forest (RF) model had the highest accuracy in the IVF/ICSI treatment. The sensitivity, F1 score, PPV, and MCC of the RF model were 0.76, 0.73, 0.80, and 0.5, respectively. These values for IUI treatment were 0.84, 0.80, 0.82, and 0.34, respectively. The BS was 0.13 and 0.15 for IVF/ICS and IUI, respectively. In addition, the estimated AUCs of the RF model for IVF/ICS and IUI were 0.73 and 0.7, respectively. Some essential features were obtained based on RF ranking for the two datasets, including age, follicle stimulation hormone, endometrial thickness, and infertility duration. The results showed a strong relationship between clinical pregnancy and a woman's age. Also, endometrial thickness and the number of follicles decreased with increasing female age in both treatments.

## Introduction

Infertility is defined as pregnancy failure after 12 months of unprotected sexual intercourse^1^. Infertility has several negative consequences for couples, such as depression, isolation, and social and personal harm^2,3^. On average, 15% of couples are reported to be infertile^4^. Various methods to treat infertility include lifestyle variation and assisted reproductive technology (ART)^5^.

International standard definitions for reporting the ART process were first presented as ICMART Glossary on ART Terminology by International Committee in 2006^6^. ARTs such as IVF and ICSI are treatments for fertilizing the egg, sperm, and embryo growth outside the body. ART has since expanded, and today, more than ten million children have been born by infertility treatments^7^. Experts choose a particular treatment depending on the conditions of the couple. Infertility treatments are generally expensive, have side effects, and are only recommended if they do not get pregnant naturally. Predictive models are suggested in medical decision-making since such comparison is a clinical challenge for gynecologists^8^. Various predictive models have been developed and evaluated until today in infertility^9,10^. For example, IVF predictive model was presented by Luke et al. [2014], which used logistic regression (LR) and stepping backward selection^11^. In addition, another model was developed by Kebbon et al.^12^. They applied an artificial neural network platform to predict live birth. Additionally, Hassen et al. [2017] suggested predictive models for IUI treatment based on multivariable logistic regression (MvLR) analysis and statistical methods, which can determine relative weights for independent features to predict pregnancy probability^13^.

Several researchers applied logistic regression to predict success rates^14–16^. Even if most prediction models are externally validated, they have limited accuracy. Therefore, the machine learning approaches were applied for predictive models that recently received more attention. Algorithms based on machine learning can strongly process medical decision-making data such as clinical predictions^17,18^. For example, Blank et al. [2019] predicted ongoing pregnancies of ≥ 11 weeks after blastocyst implantation in a fresh day-5 single-embryo transfer IVF cycle and compared it with a MvLR^19^. Furthermore, Lijuo et al. [2020] compared six machine learning models to predict fetal heart rate in IVF treatment^20^.

We tried to use the machine learning perspective for predictive models and compared the performance of these tools compared to logistic regression for our infertility datasets. To the best of our knowledge, this is the first comparison of well-known machine learning models to predict clinical pregnancy for IUI and IVF/ICSI treatment at the same time.

## Methods

### Data collection and study variables

Two infertility centers, one public infertility center owned by the University of Medical Sciences and one private infertility center in Mashhad, Iran, participated in the retrospective study. Inclusion criteria are data were collected from all infertile couples who completed their IVF/ICSI cycle in these centers. Also, data from the couples who needed sperm or donor eggs or surrogacy uterus were excluded. Only the first three cycles of treatment were considered. Also, all infertile couples who left their treatment cycle incomplete or more than 50% of the required clinical factors missed were considered exclusion criteria. The model output was clinical pregnancy, i.e., ultra-sonographic visualization of one or more gestational sacs or definitive clinical signs of pregnancy. The predictive features of each treatment were obtained systematically^21^. Finally, the data related to 17 features of patients under IUI and 38 features for patients under IVF/ICSI were collected (can be seen in Supplementary Tables 1 and 2 with more details). This study was approved by the Institutional Review Board (IRB code: IR.MUMS.MEDICAL.REC.1399.060.) of Mashhad University of Medical Sciences. We have been obtained informed and free written consent. We confirm that all methods were performed following the relevant guidelines and regulations.

A total of 1000 IVF/ICSI cycles (Cycle means the number of treatment courses under IVF/ICSI) and 1485 IUI cycles were collected to predict pregnancy. Clinical Pregnancy Rates (CPR) were 32.7% and 18.04% in IVF/ICSI and IUI treatments, respectively. In the IVF/ICSI dataset, 75.5% of the couples had primary infertility. The major cause of infertility was male factors (45.5%). Furthermore, the average infertility duration was 6.09 years. In this dataset, about 22.2% of the couples had prior treatment by IVF/ICSI. The IVF/ICSI dataset contains 38 features, of which, except for eight factors, the rest do not have a significant effect on predicting the occurrence of clinical pregnancy (Supplementary Table 1).

A total of 1485 IUI treatment cycles were included, and the most cause of infertility was 27.25% of unexplained causes. Of the 1196 couples, 72.31% had primary infertility and an average infertility duration of 4.36 years. Also, in the IUI dataset containing 17 features, only nine factors were significantly associated with the occurrence of clinical pregnancy (Supplementary Table 2). FSH, an essential hormone for the growth and function of ovaries and testicles, is one of the critical features in chances of pregnancy success after IVF and IUI^22^. We consider it Basal day 3 FSH (FSH) assessed on the third day of the cycle. In the IVF/ICSI cases, the FSH of 76.8% of the women was 3 to 10 mIU/ml, of which 80% were under 35 years. Furthermore, In the IUI cases, the FSH of 77% of women was 3 to 10 mIU/ml, of which 87% were under 35.

Moreover, in the IUI treatment, the number of follicles is evaluated by performing an ultrasound on the second day of the cycle. If there is no cyst in the ovary and the endometrial thickness is appropriate, medications such as GnRH, including FSH, Clomiphene Citrate, and Letrozole, are prescribed. After six days, the follicles are evaluated, and if they are more than 16 mm, the HCG is injected. In IVF/ICSI method, the sperm and egg are taken from the couple, and after fertilization and embryo formation in the laboratory, the embryos are transferred into the uterus. The ovarian stimulation protocol is administered after the first ultrasound on the second day of the cycle. After initial tests, follicle-stimulating hormone (FSH) drugs (including Letrozole, Clomiphene Citrate, Cinal F, Cetrotide, Superfact, HMG) are prescribed in this method for follicle growth. After stimulation of ovaries, follicles more than 17 mm thickness, suitable for endometrium, ovulation-stimulating drug (HCG) are injected, and ovulation occurs about 36 h later. Meanwhile, the sperm with the highest quality is selected, and fertilization is performed in embryonic culture media. After 16 to 20 h, the signs of fertilization are examined.

### Pre-processing and missing data

Using a mean or a fixed number is a traditional way to fill the missing values. For this purpose, a more precise method is prediction models, such as regression and classification. The missing values in this data set were small (3.7% for IUI and 4.09% for IVF/ICSI).

Multi-Level Perceptron (MLP) was used to predict the missing values. MLP provides better results than classic imputation strategies for missing values^23^. Therefore, despite the difference in data (noise), acceptable values were obtained for the missing values. Then, 80 and 20% of the dataset were randomly selected for training and testing, respectively. The k-fold cross-validation method with k = 10 was used to evaluate the model. Cross-validation is a method used to approximate the performance of machine learning models. It is applied to avoid overfitting problems in the predictive model and is suitable for small datasets. In cross-validation, a fixed number (named folds) of the data, run the analysis on each fold, and then average error approximate.

### Models construction

**Figure 1:**
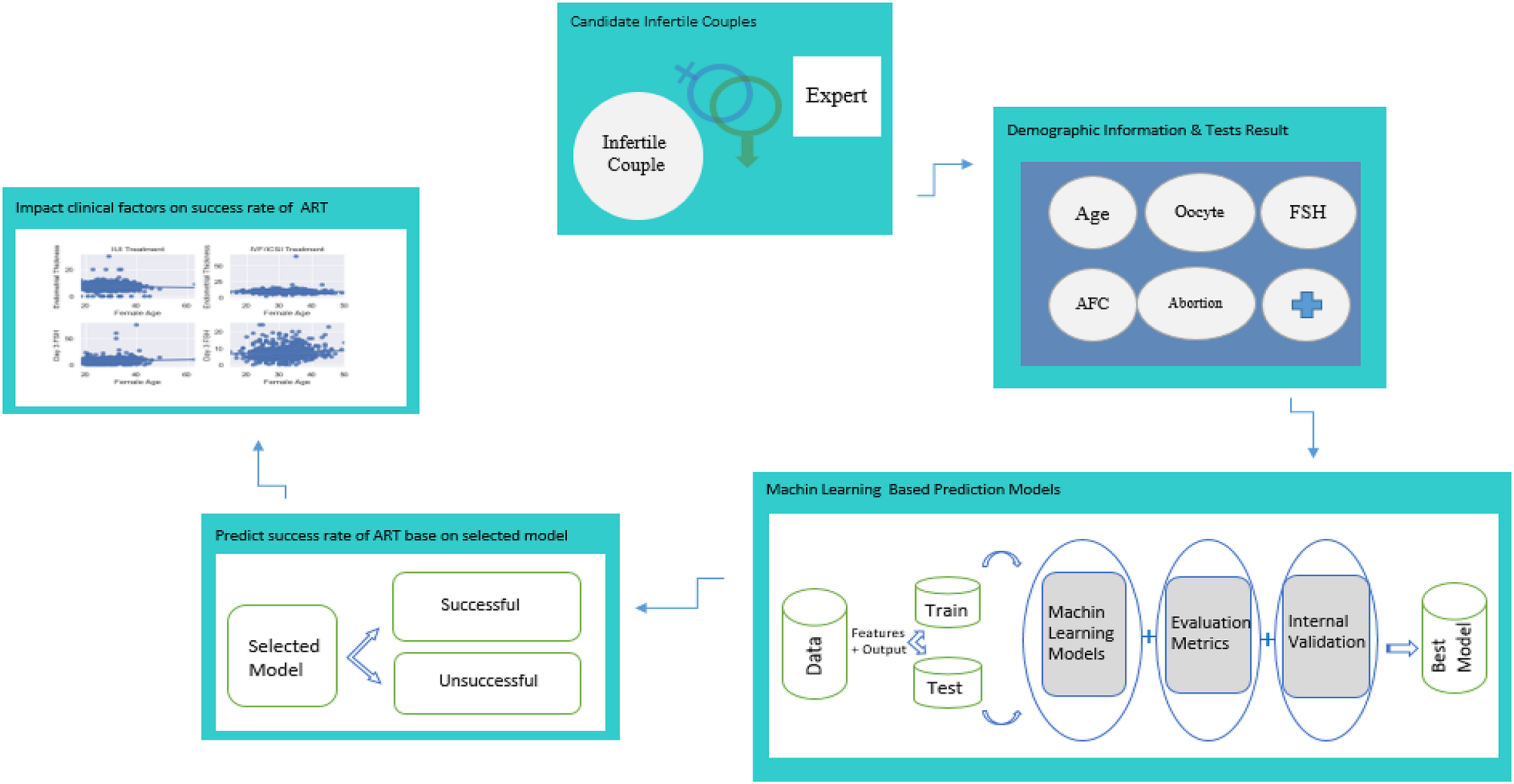
Graphical abstract of our study.

Classification and regression are the two most important machine learning algorithms. Although Logistic Regression (LR) is a standard supervised classification algorithm, six well-known machine learning algorithms include LR, Random Forest (RF), k-Nearest Neighbors (KNN), Artificial Neural Network (ANN), Support Vector Machine (SVM), and Gradient Naïve Bayes (GNB) were considered for making prediction models. The algorithms were applied to predict the success rate of IUI and IVF/ICSI treatments by using Python (version 3.8.). Random search with cross-validation was chosen to optimize hyperparameters in classifiers^24^. The study roadmap is shown as a graphical abstract in Fig. 1.

### Evaluation metrics

#### Metric

The outcome in our predictive model was binary, successful, and unsuccessful. The most common criteria were used to evaluate the performance of the models. Suppose the total number of samples is N, the confusion matrix, which divides samples into four sections named True Positive cases (TP), True Negative cases (TN), False Positive cases (FP), and False Negative cases (FN) (See Supplementary Table 3a). In that case, evaluation metrics are defined in Supplementary Table 3b. The measures were used include accuracy (the correct number of outputs predicted), precision (the ratio of positives that are correctly predicted to the total values that are correctly predicted), recall (the ratio of the positive values predicted by the model to all the actual positive values) and F1-score which is the average harmonic value of precision and recall.

##### Brier score

The model's overall accuracy was assessed using the Brier (BS) score. This criterion represents the predicted, and actual values squared difference, including 0 to 1. The zero value of 0 indicates a model with an excellent prediction difference, while 1 shows an entirely wrong prediction^25^.

##### Matthew correlation coefficient

Because the case data are unbalanced, the Matthew correlation coefficient (MCC) is used better to evaluate the performance of the methods^26^. This criterion is suitable for binary classification, and it is based on positive and negative samples that are calculated as follows:

1 $$MCC = \frac{TP*TN - FN*FP}{{\sqrt {\left( {TP + FP} \right)\left( {TP + FN} \right)\left( {TN + FP} \right)\left( {TN + FN } \right)} }}$$

The values of this criterion lie in the range − 1 to 1 so that the closest value to 1 indicates the better the model predicts.

##### Receiver operating characteristics

Receptor operational characteristics (ROC) charts evaluate classification models and control performance^27^. The range of changes in this curve is between 0 and 1. Additionally, the value of the area under the ROC curve was measured as AUC. The AUC value varies between 0.5 and 1, where 0.5 describes unfavorable, and 1 describes superior performance for the classifier. Then, Delong's method was applied to compare the AUCs of the models^28^.

## Results

Six classification models were provided to predict infertility success rates. These models were evaluated based on accuracy, AUC, PPV, F1, and sensitivity criteria. As shown in Table 1, the RF model has the best performance in predicting the success rate in IVF/ICSI treatment by obtaining the highest accuracy (0.76). Furthermore, this model obtained higher values in other criteria. The RF model has the best performance in the IUI dataset by obtaining 0.84 for accuracy. Other criteria in this model also had equal or higher performance. Also, the MCC measure in the RF model is highest with 0.5 and 0.29 for IVF/ICSI and IUI, respectively.

**Table 1:** Comparison among results of different prediction models with clinical pregnancy in IVF/ICS and IUI treatment.

Model	Treatment	PPV	Sensitivity	F1	Accuracy	AUC	BS	MCC
LR	IVF/ICSI IUI	0.75 0.84	0.69 0.84	0.60 0.77	0.68 0.83	0.69 0.68	0.31 0.16	0.26 0.04
RF	IVF/ICSI IUI	0.80 0.84	0.76 0.84	0.73 0.8	0.76 0.84	0.73 0.70	0.13 0.15	0.50 0.34
SVM	IVF/ICSI IUI	0.42 0.68	0.65 0.82	0.51 0.75	0.64 0.82	0.5 0.64	0.35 0.17	0.00 0.00
MLP	IVF/ICSI IUI	0.72 0.68	0.69 0.82	0.63 0.75	0.69 0.82	0.63 0.68	0.30 0.175	0.32 0.14
KNN	IVF/ICSI IUI	0.74 0.77	0.74 0.81	0.71 0.78	0.74 0.81	0.50 0.64	0.23 0.15	0.06 0.24
GNB	IVF/ICSI IUI	0.67 0.72	0.70 0.68	0.70 0.75	0.69 0.75	0.67 0.67	0.39 0.21	0.16 0.17

PPV, Positive Predictive Value; AUC, Area Under ROC Curve; BS, Brier Score, MLP, Multi-Level Perceptron; SVM, Support Vector Machine; LR, Logistic Regression; RF, Random Forest; KNN, k-Nearest Neighbor; GNB, Gaussian Naïve Bayes.

**Figure 2:**
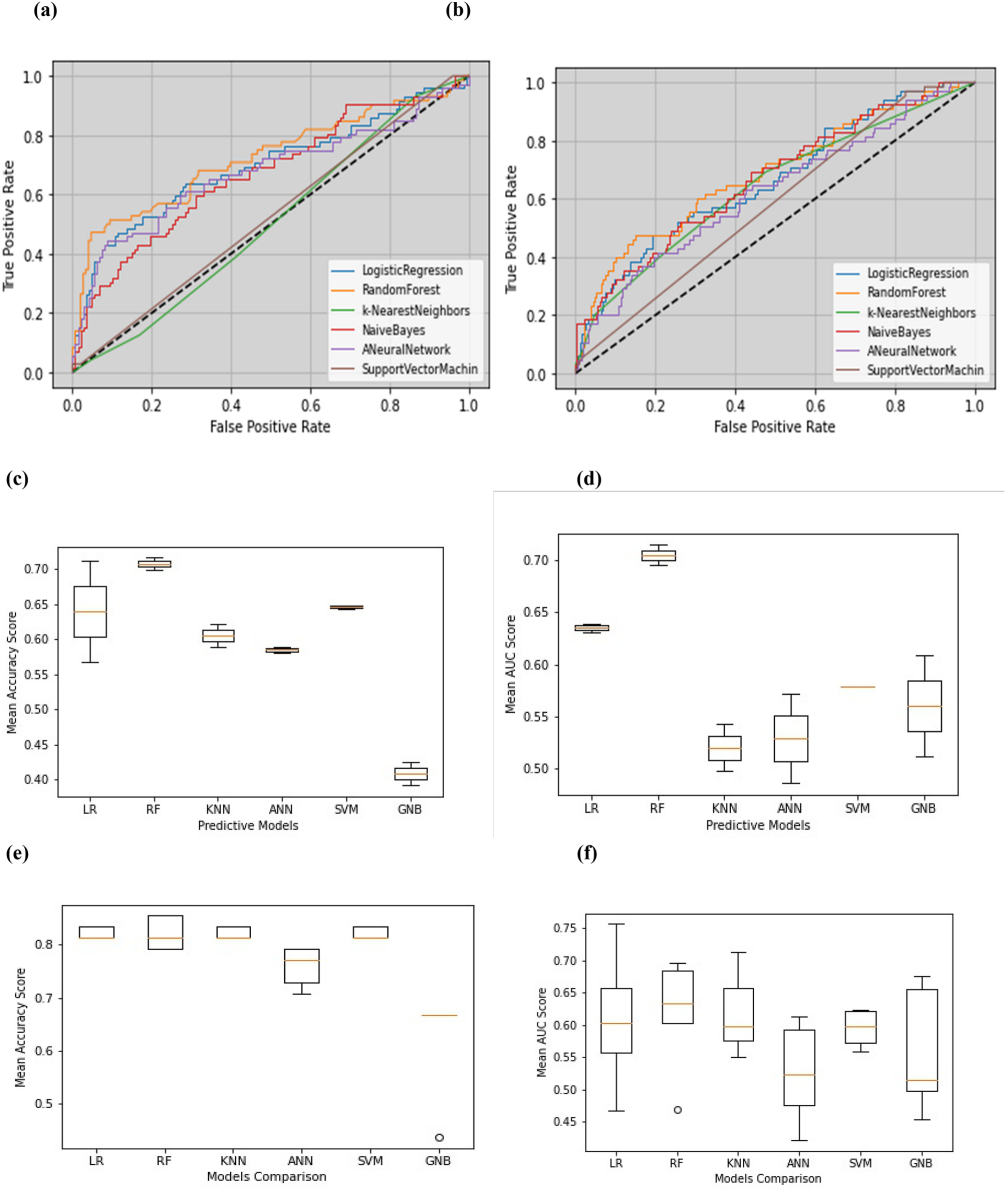
ROC Curves for IVF/ICSI and IUI. (**a**) ROC Curves by the models for IVF/ICSI; (**b**) ROC Curves by the models for IUI; Comparison among cross-validation process based on the accuracy (**c**) for IVF/ICSI (**e**) for IUI and AUC score (**d**) for IVF/ICSI and (**f**) for IUI.

ROC plot was used to show the performance of the model intuitively. Figure 2a shows ROC plots for the presented models based on IVF/ICSI dataset, respectively. As can be seen, the RF model has a higher AUC (0.73) than other models. These diagrams for IUI treatment are shown in section (b) from Fig. 2. The RF model has the best AUC (0.70) in this data set. No significant difference was observed among AUCs regarding the *p* value (*p* > 0.05).

In addition, we added new results for a better comparison of the methods based on AUC and accuracy scores in the cross-validation process for each treatment. As shown in Fig. 2c-f, the best mean accuracy and AUC scores in the cross-validation process are obtained for the RF model in IVF/ICSI and IUI treatment.

### Correlation between the factors

Supplementary Tables 1 and 2 present the contribution of 38 candidate factors of IVF/ICSI treatment and 17 candidate factors of IUI treatment, respectively. We analyzed the significance between the candidate factors and the clinical pregnancy by *t* test.

**Figure 3:**
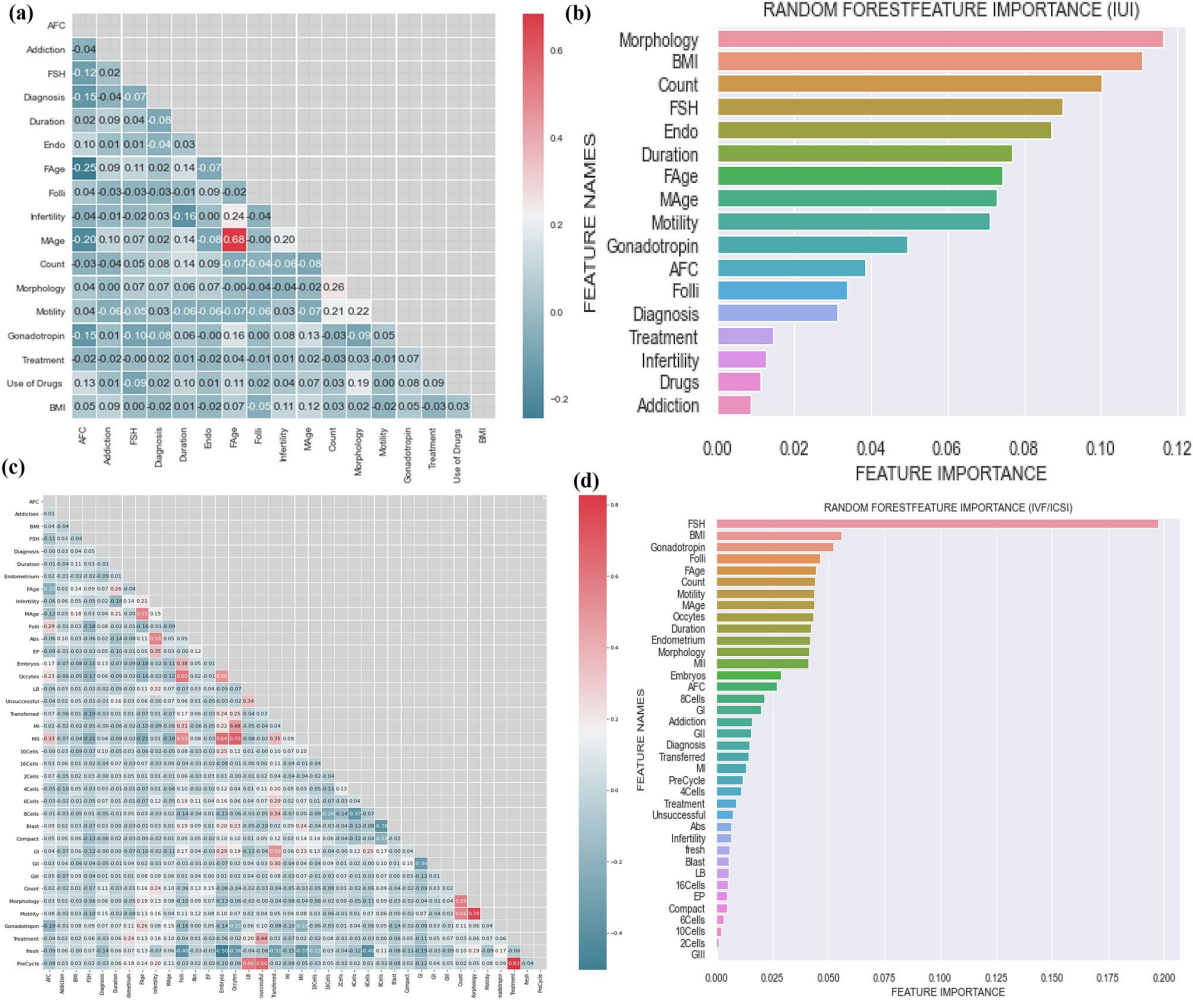
Correlation and Important Features in IVF/ICSI and IUI Treatment, (**a**) Correlation of features in IUI dataset, (**b**) Important features in IUI dataset, (**c**) Correlation of features in IVF/ICSI dataset, (**d**) Important features in IVF/ICSI dataset. The figures were drawn by using python platform version 3.8.

Predictive features have been used in predictive models that affect the success rate of infertility treatment. The correlation matrix shows the relationship between these features. The heat map was used to demonstrate this Correlation better. The Correlation calculated by Pearson function and threshold is 0.85. If there is a high correlation between two features (higher than a threshold), the corresponding cell is red. In both treatments, the predictive features are not highly correlated. The value inside each cell indicates the degree of Correlation between the two features (See Fig. 3a,c). A random forest ranking is used to detect the importance of the clinical factors for each treatment method (Fig. 3b,d).

According to the Fig. 3b,d, characteristics such as FSH, BMI, female age, endometrial, duration, gonadotropin, and semen analysis (count, motility, and morphology) are some of the essential features in IVF/ICSI and IUI treatments. In addition, the number of follicles is a common feature that plays a crucial role in the IVF treatment, but not in the IUI treatment method. Further, the number of oocytes is an important non-common feature in IVF/ICSI treatment. All the figures were drawn by python 3.8.

### Effect of the proposed model

**Figure 4:**
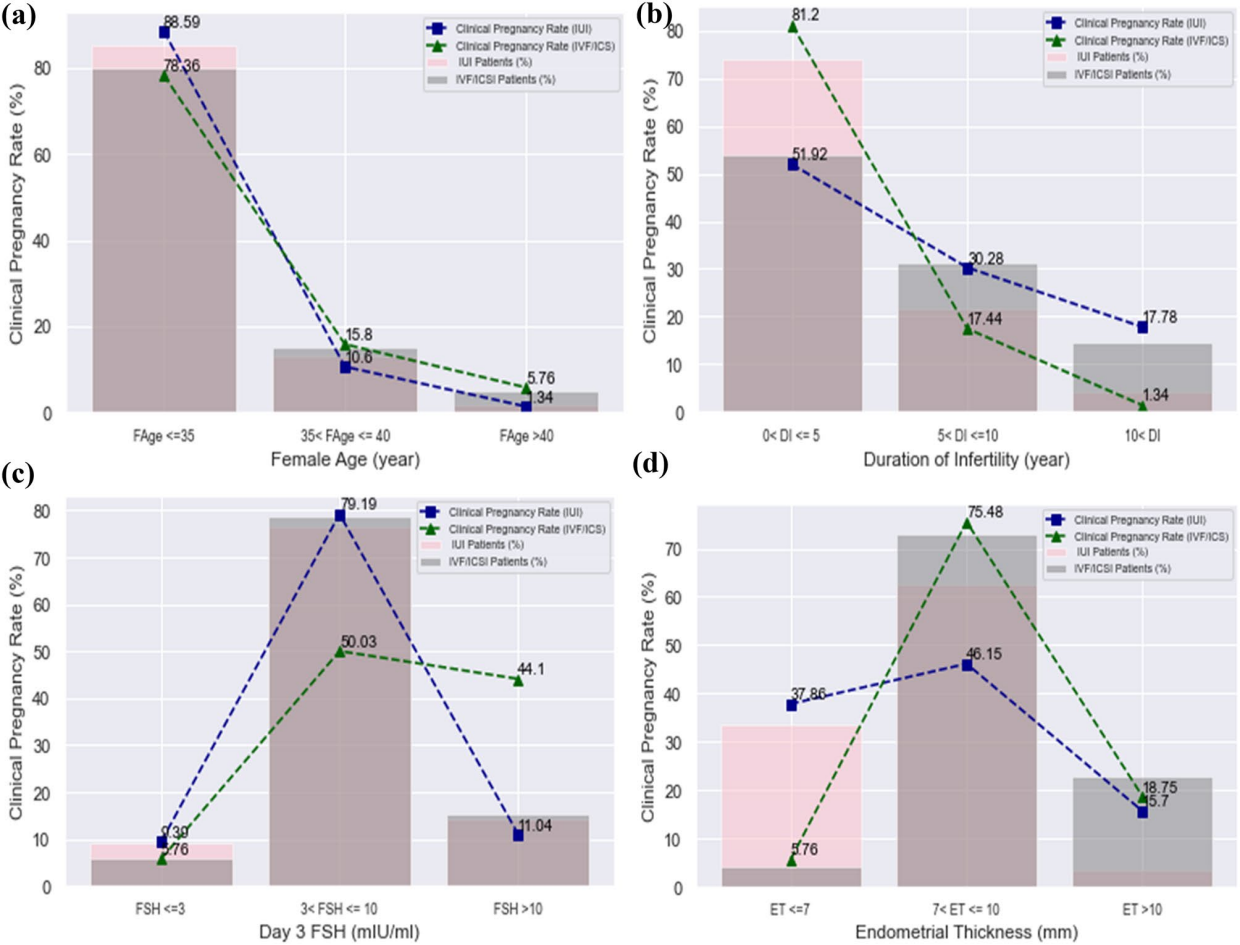
Impact of essential features on CPR based on results of RF model. (**a**) Comparison between CPR and Female Age for IVF/ICSI and IUI datasets, (**b**) Comparison between CPR and Duration of Infertility, (**c**) Comparison between CPR and FSH, (**d**) Comparison between CPR and Endometrial Thickness.

The impact of notable important features on CPR was presented using the RF model. Female age is an essential feature in infertility treatment methods. The success rate decreases by increasing female age. This relevance can be seen by the RF model for each treatment in Fig. 4a.

The effect of infertility duration on clinical pregnancy was examined. Higher infertility duration can decrease the success rate^29^. This relationship can be seen for the treatment methods in Fig. 4b. The cut-off FSH in 3 to 10 mIU/ml has the highest CPR (Fig. 4c). The level of this hormone varies according to individual characteristics and reduces the success rate of IVF/ICSI in women younger than 35 for values higher than 8. ET is another essential feature in the success of infertility treatment^30^. Endometrial thicknesses less than 10 mm in 77.11% of the IVF/ICSI dataset are visible. This feature is in the range of 7–10 mm in 62.65% of the IUI data set. The CPR increases in both treatments by increasing Endometrial thickness up to 10 mm. After this point, the clinical pregnancy rate decreases (See Fig. 4d).

### Impact of clinical factors

**Figure 5:**
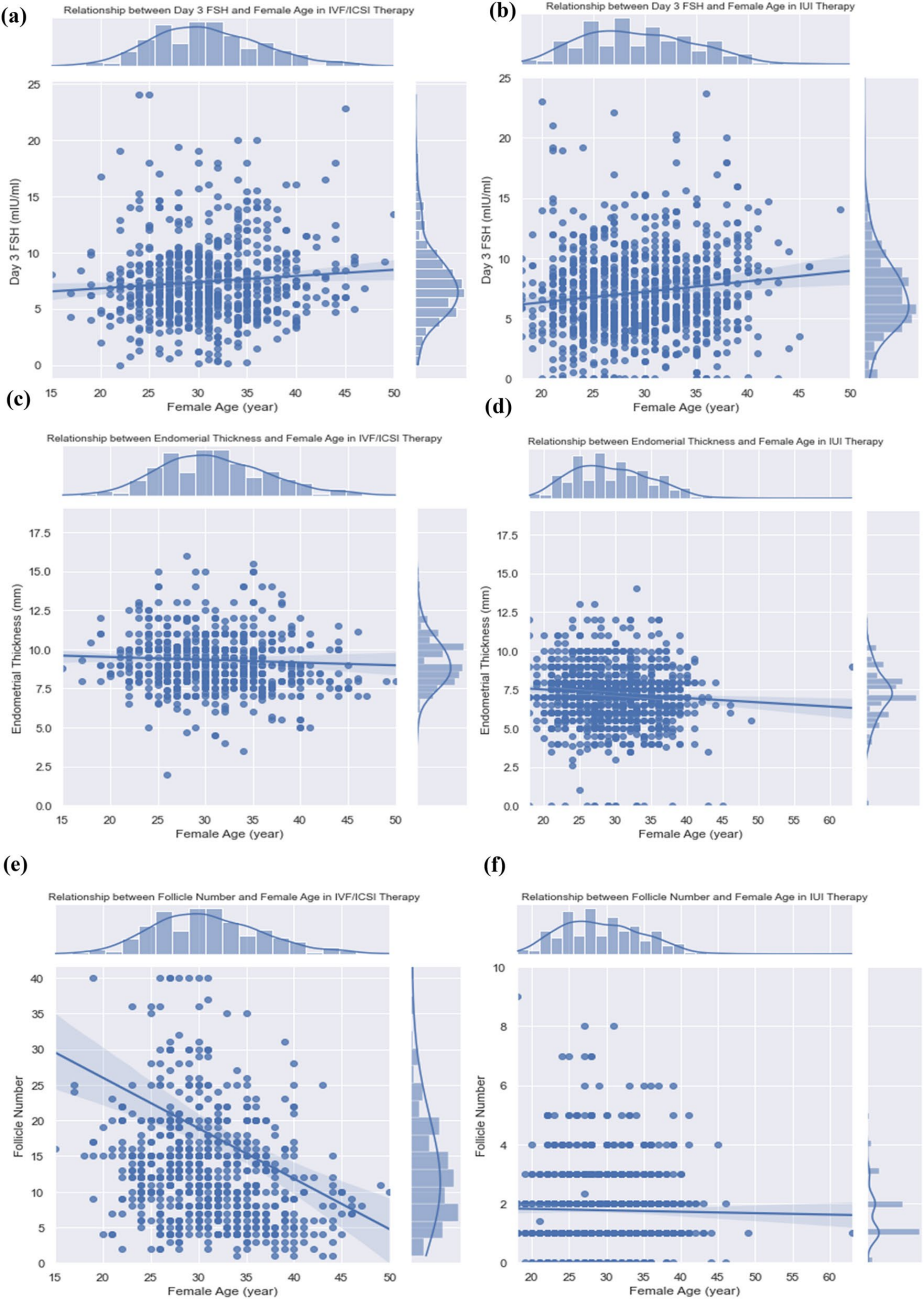
Relationship of Female Age with FHS for (**e**) IVF/ICSI, (**f**) IUI; Relationship of Female Age with Endometrial Thickness for (**g**) IVF/ICSI, (**h**) IUI; Relationship of Female Age with Number of Follicles > 16 mm for (**i**) IVF/ICSI, (**j**) IUI.

The effect of some clinical factors on female age is evaluated in this section. For this purpose, connected distribution diagrams are used. The diagrams show the scatter plot and the histogram data to display detailed information based on bivariate distributions. As can be seen in Fig. 5a,b, the FSH increases by increasing women's age in both treatments, although this increase is steeper in the IUI treatment. FSH is dispersed in both methods, but this dispersion has the highest density between 5 and 10 mIU/ml. Figure 5c,d show the relationship between women's age and endometrial thickness in the two treatments. The endometrial thickness has an almost decreased trend by increasing age. In addition, in the IVF/ICSI method, endometrial thickness is limited to 9–11 mm. These changes are similar in IUI but have a lower slope, and endometrial thickness lies in 7–8 mm for women under 40. The scatter in both figures is negligible. Figure 5e,f demonstrate the relationship between the number of follicles > 16 mm and the woman's age in the two treatments. Overall, the number of follicles > 16 mm decreased by increasing the woman's age, although this descending slope is more in the IVF/ICSI treatment method.

To get more follicles > 16 mm, minor age in women is needed for each treatment, although this slope is more in the IVF/ICSI treatment method. Dispersion is considerable in the IVF/ICSI treatment and mainly involves between 5 and 40 follicles > 16 mm, while it is much less in IUI treatment and mainly between 1 to 5 follicles > 16 mm.

### Comparison treatment methods

This section presents the comparison between common factors in the two treatment groups. Common factors for each of the treatments in patients with positive clinical pregnancy are shown in Table 2. As can be seen, the most common factors in the two treatment groups are significantly different. For example, women's age and FSH differ among patients with a successful pregnancy in each treatment method. As shown in Table 2, in couples with a successful pregnancy, the mean ages of women were 28.83 and 31.37, and the mean FSH levels were 6.37 and 9.23 (mIU/ml), for IUI and IVF/ICSI, respectively.

**Table 2:** Clinical characteristics of couples undergoing IVF/ICSI and IUI with common factors.

Characteristic	Successful (IVF/ICSI) (N = 240)	Successful (IUI) (N = 216)	*p* value
Age			
Female	31.3 ± 5.5	28.8 ± 5.2	< 0.001a*
Male	35.4 ± 9.9	32.4 ± 5.0	0.018 a*
AFC	10.1 ± 5.4	11.58 ± 5.5	0.141 a
FSH (mIU/ml)	9.2 ± 4.4	6.3 ± 3.3	0.005 a*
Addiction			
Non	139 (57.96%)	202 (93.51%)	0.213c
Smoke	33 (13.75%)	4 (1.85%)	
Alcohol	15 (6.2%)	1 (0.46%)	
Narcotic	53 (22.9%)	7 (3.24%)	
		2 (0.92%)	
Diagnosis			0.0076c*
Male Factor	100 (41.66%)	42 (19.44%)	
Female Factor	70 (29.1%)	9 (4.16%)	
Unexplained	22 (9.1%)	53 (24.53%)	
Mix	48 (20%)	51 (23.67%)	
Duration of Infertility (year)	6.1 ± 4.7	3.52 ± 2.67	0.0074 a*
Endometrial Thickness (mm)	9.0 ± 1.8	6.95 ± 2.19	
Follicle Number	15.3 ± 22.3	1.81 ± 1.22	< 0.001 a*
Infertility			0.09
Primary	185 (77.08%)	147 (68.05%)	
Secondary	55 (22.9%)	69 (31.9%)	
Sperm			
Count	54.0 ± 49.8	77.9 ± 46.0	0.0064a*
Motility	35.2 ± 22.8	39.8 ± 21.0	0.0049 a*
Morphology	32.3 ± 22.8	50.3 ± 14.5	0.0051 a*
Total Gonadotropin Dose	32.9 ± 13.7	4.9 ± 3.8	0.027 a*
Treatment Cycle Number			
Cycle1	180 (75%)	184 (85.18%)	0.074
Cycle 2	49 (20.4%)	26 (12.03%)	
Cycle 3	11 (4.5%)	6 (2.7%)	
BMI	25.4 ± 4.4	25.6 ± 3.7	0.03 a*

AFC, Antral Follicle Count; FSH, *Follicle Stimulating* Hormone; BMI, Body Mass Index, Mean ± Standard Division for continues and N (%), percent of the number of couples for categorical variables are presented.

*Significant features (*p* value < 0.05).

^a^Examined via Student's *t* test.

^b^Examined via Fisher's exact test.

^c^Examined via Chi-square test.

## Discussion

The present retrospective study of IVF/ICSI and IUI treatments provided a predictive model for calculating the CPR of each treatment. Machine learning algorithms with strong data processing capabilities are more insightful methodologies for infertility data and have been considered in clinical decision-making and medicine studies by researchers^19,20,31^.

This study implemented and evaluated machine learning models to predict CPR for treatments. The results showed that the random forest outperformed other algorithms, and it had a strong relationship with the CPR, women's age, and infertility duration. The obtained F1 score, BSs, and AUCs it yielded with CPR for IVF/ICSI, IUI treatments (0.73, 0.13 and 0.80, 0.15 and 0.65, 0.70, respectively) showed its excellent performance. This algorithm ranks the features by calculating Gini Index in each branch. The results showed that clinical factors such as FSH, women's age, endometrial thickness, and duration were some of the most important common features in IVF/ICSI and IUI treatments. Previous studies have shown that women's age, infertility duration, and FSH are well-known predictors^12,32,33^. Using the essential features showed that CPR decreased by increasing the age and duration of infertility.

Endometrial thickness (ET) is an important predictive factor for pregnancy success. Several studies have been performed to determine ET. However, more studies are needed to be done, but some studies showed a suitable cut-off for this factor above 7 mm in IVF treatment^34^. Although the cut-off for this factor has been reported as 10.5 and 13.5 mm in IUI treatment^35^, no significant association was reported for IUI treatment^36^. The present study results showed that the cut-off for ET with the highest CPR lies in the range of 7–10 mm for IVF/ICSI and IUI treatment. FSH was another significant predictor of the success of infertility treatments. Previous studies indicated that although the cut-off of 10 IU/ml for this factor led to the highest CPR and live birth for IVF treatment, no significant association was found between pregnancy and FSH levels^37^. In another study, there was a significant association between CPR and FSH values 9 IU/L or higher in IUI treatment^38^.

This study showed the cut-off (highest CPR) for FSH in 3 to 10 mIU/ml. As shown in Table 2, patients with unexplained infertility have a higher chance of success in IUI treatment. Additionally, if the cause of male infertility or unfavorable semen analysis conditions, the success chance of the IVF method is higher. Although the mean AFC in the IVF method is significantly higher than the IUI method, the high average value for this factor cannot lead to a higher chance of pregnancy by the IVF method (Table 2). Furthermore, since the ovarian function is weaker in older women, most older couples need more follicles, therefore as they are more likely to be treated with IVF, the average number of follicles is significantly higher than the IUI method (15.33 vs. 1.88).

Although there is a significant relationship between successful and unsuccessful groups in the IVF method (15.33 vs. 19.6), high values of this factor can lead to loss of pregnancy chance. Furthermore, the comparison of data Table 2 and Supplementary Table 2 indicate that the average duration of infertility in the IUI treatment is significantly less than the average value of this factor in the IVF treatment. Based on Supplementary Table 2, it can be concluded that lower than average values of this factor can lead to higher success chances in the IUI method, which is in contrast to the IVF method.

Furthermore, having a higher chance of getting pregnant by IVF requires a higher FSH level (average greater than 8). According to Supplementary Table 1, high levels of this hormone are significantly related to increasing the pregnancy chance in this method. In addition, lower levels of this hormone (average 6.37) are significantly related to the success of the IUI method. The mean endometrial thickness in IVF is greater than IUI. Although there is a significant difference in the mean of this factor in the two treatments (Table 2), it cannot be said that high endometrial thicknesses can lead to a higher success chance of the IVF method (Supplementary Table 1).

What makes this study different from other studies is the aim of this study to predict CPR based on a machine learning approach in different methods of infertility treatment simultaneously. Additionally, BS was considered for the accurate evaluation of classifiers such as GNB, SVM, KNN, and ANN were compared with RF. Moreover, CPR was evaluated in IVF/ICSI and IUI treatments.

Our data set was class-imbalanced (i.e., the main class of interest is rare). In addition to specificity and sensitivity, the MCC criterion, which is suitable for class-imbalanced data sets, has been used. The MCC criterion showed that the RF model had the best prediction performance.

The RF model also accurately predicts 97% of failed pregnancies and about 0.5% of successful pregnancies on the test data. It is important to note that although the prediction rate in successful samples by RF is not significant, it is a problem in the other models so that the prediction rate in the successful group is lower than 50%. It is due to the type of the data.

The study has some limitations and precautions. The data were collected from only two infertility centers from one city. Therefore, it is suggested that data be collected from several centers in different geographical locations, and external verification will have better performance and reliability. In addition, the size of the dataset used was not significant, especially in successful samples, which is considered a precaution in this study.

Future research topics could include selecting the weight corresponding to each feature and determining model parameters using meta-heuristic algorithms and fuzzy theory for ranking. Furthermore, due to the type of infertility data, which is often unbalanced, it is recommended to use data smoothing and binning methods in pre-processing step and the leave-one-out method in the validation phase.

## Conclusion

In this study, machine learning algorithms were applied in pre-processing the datasets and creating models using data from infertile couples treated by IVF/ICSI and IUI. It first compares machine learning predictive models for IUI and IVF/ICSI treatment. The results showed that the RF had higher accuracy among the treatment methods. Some essential features were obtained based on RF ranking for the two datasets, including age, follicle stimulation hormone, endometrial thickness, and infertility duration. The results showed a strong relationship between clinical pregnancy and woman's age infertility duration. Also, endometrial thickness and the number of follicles decreased with increasing female age in both treatments. Furthermore, sperm morphology and follicle stimulation hormone were the essential factors in the IUI and IVF/ICSI treatment methods based on the RF model.

## Supplementary Information

Supplementary Information 1.

Supplementary Information 2.
