# Supplementary material for: Internal validation and comparison of predictive models to determine success rate of infertility treatments: a retrospective study of 2485 cycles
Source: Sci Rep. 2022 May 4;12:7216. doi: 10.1038/s41598-022-10902-9 (PMC9068696; doi:10.1038/s41598-022-10902-9)
Supplement: Supplementary file 1 — Supplementary Information 1. [file 41598_2022_10902_MOESM1_ESM.docx]

We used six prediction models, the details of which are as following Tables.

Supplementary Table 4: Hyper-Parameter Setting are obtained by Random Search algorithms for the models in IVF/ICSI treatment

| **Name of Methods in IVF/ICSI** | **Hyper parameters Setting** | | |
| --- | --- | --- | --- |
| Random Forest | n_estimators=1500 | criterion=entropy | max_features=’sqrt’ |
| Logistic Regression | solver=‘saga | max_iter=100 | multi_class='auto' |
| Gaussian Naïve Bayes | There is no setting for GNB classifier | | |
| Support Vector Machine | kernel='rbf' | C=1.0 | probability=True |
| Artificial Neural Network | hidden_layer_sizes=10 | activation='logidtic' | max_iter=34 |
| K -Nearest Neighbors | n_neighbors=10 | Algorithm=‘auto’ | P=8 |
|  |  |  |  |

Supplementary Table 5: Hyper-Parameter Setting are obtained by Random Search algorithms for the models in IUI treatment

| **Name of Methods in**  **IUI** | **Hyper parameters Setting** | | |
| --- | --- | --- | --- |
| Random Forest | n_estimators=1067 | criterion=entropy | max_features =10 |
| Logistic Regression | solver=‘saga’ | max_iter=100 | multi_class=multinomial' |
| Gaussian Naïve Bayes | There is no setting for GNB classifier | | |
| Support Vector Machine | kernel='rbf' | probability=True | C=1.0 |
| Artificial Neural Network | hidden_layer_sizes=15 | activation='logidtic' | max_iter=70 |
| K -Nearest Neighbors | n_neighbors=10 | Algorithm=‘auto’ | P=10 |
|  |  |  |  |

In addition, we show results of methods as comparison accuracy and roc_auc for each methods in IVF/ICSI treatment as follows:

**
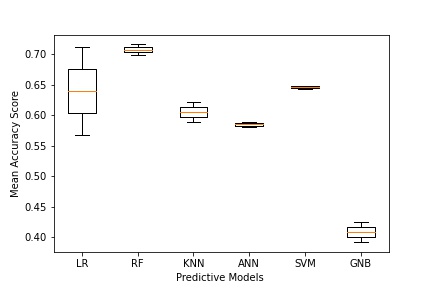
**
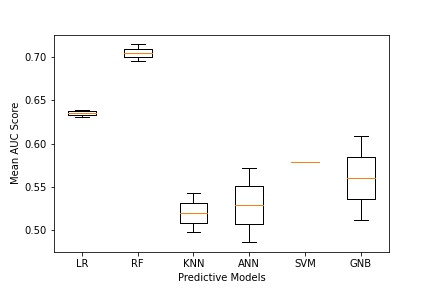


Supplementary Figure 1: Comparison of models based on Supplementary Figure 2: Comparison of each methods based on

accuracy in IVF/ICSI treatment AUC value in IVF/ICSI treatment


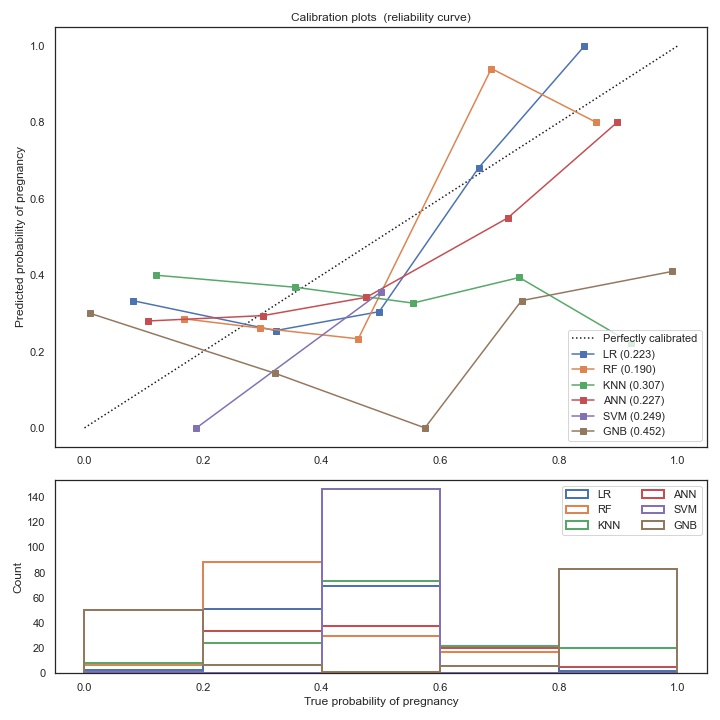


Supplementary Figure 3: Calibration plots (reliability curve) for models in IVF/ICSI treatment

We show confusion matrix for each of models based on test data with 20% of total sample size (147 of 733) for IVF/ICSI treatment in detailed as follows:


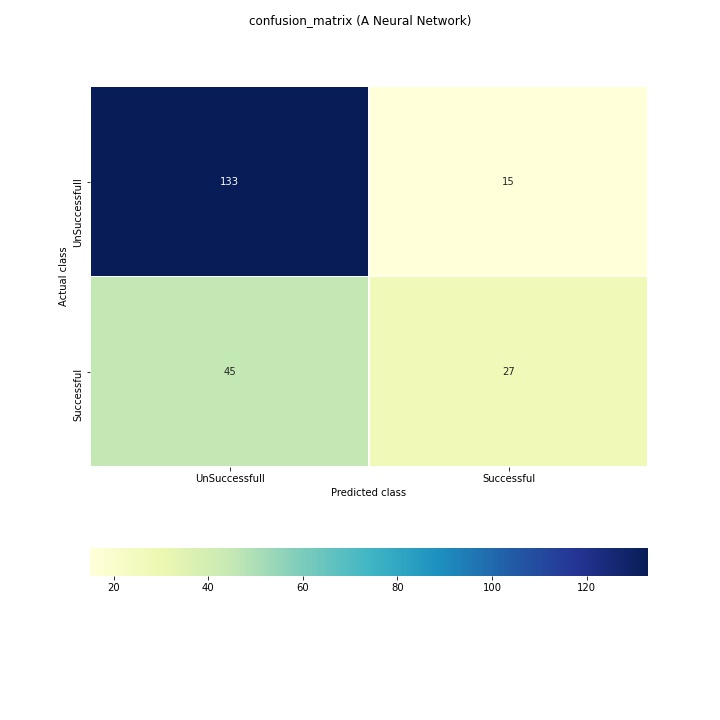

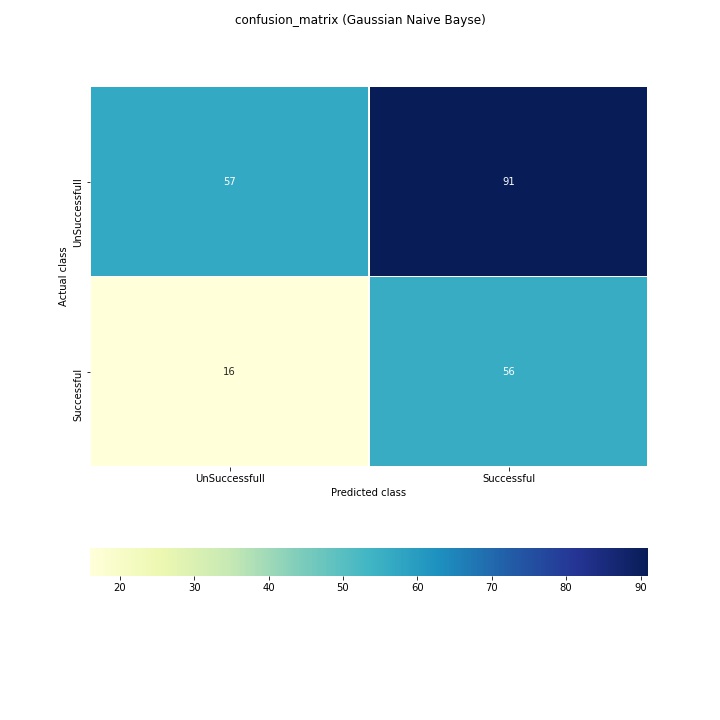

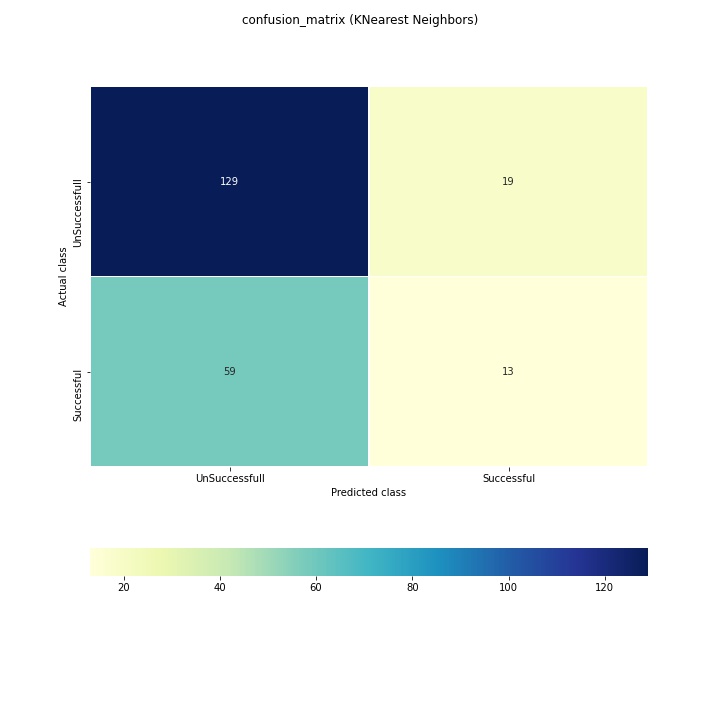


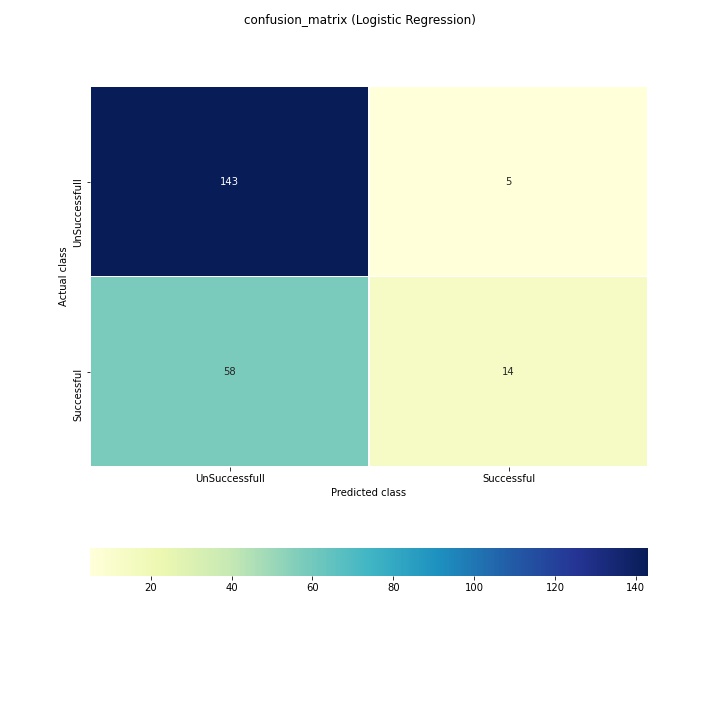

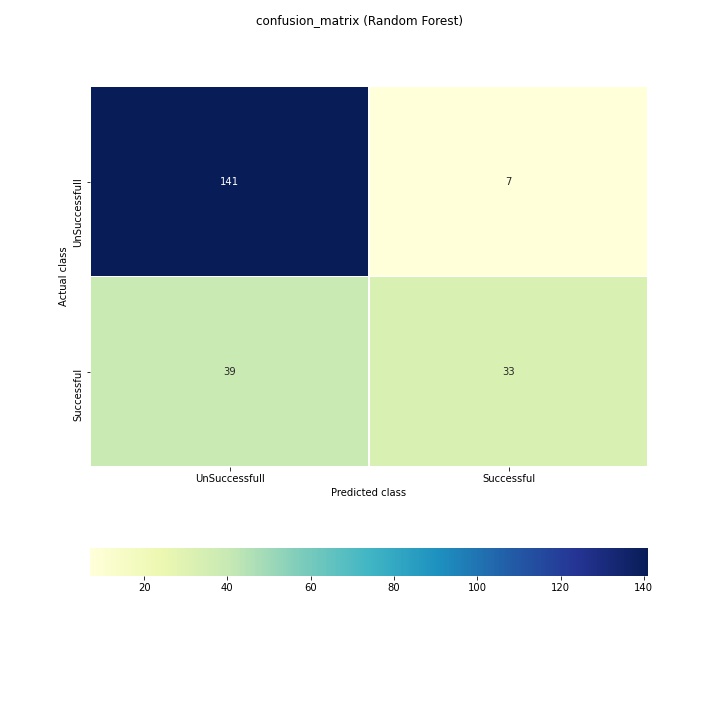

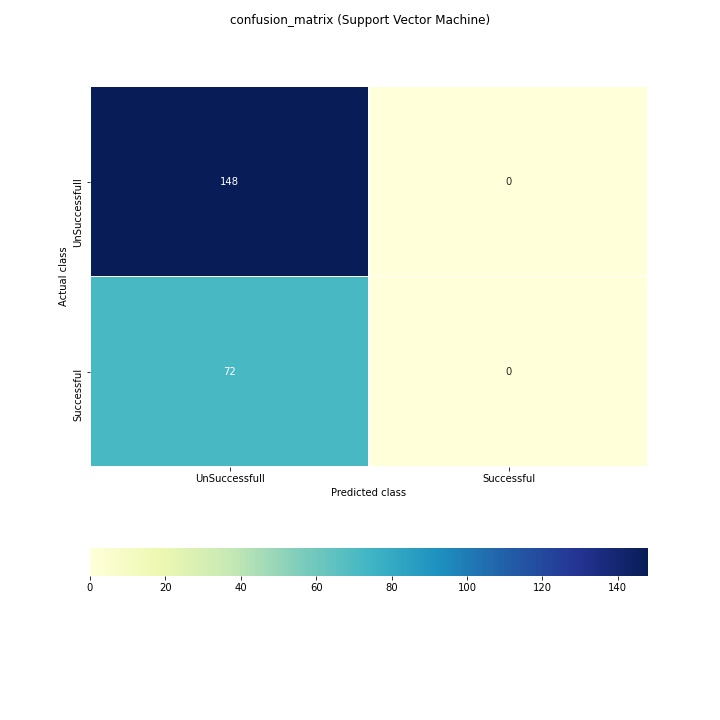


Supplementary Figure 4: Comparison among confusion matrix for models in IVF/ICSI treatment

Also, this results are prepared for IUI treatment as follows:


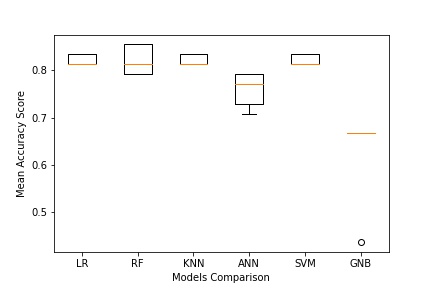

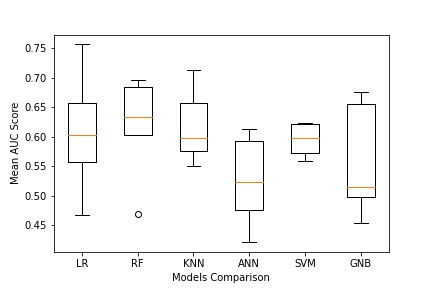


Supplementary Figure 5: Comparison of models based on Supplementary Figure 6: Comparison of each methods based on

accuracy in IUI treatment AUC value in IUI treatment
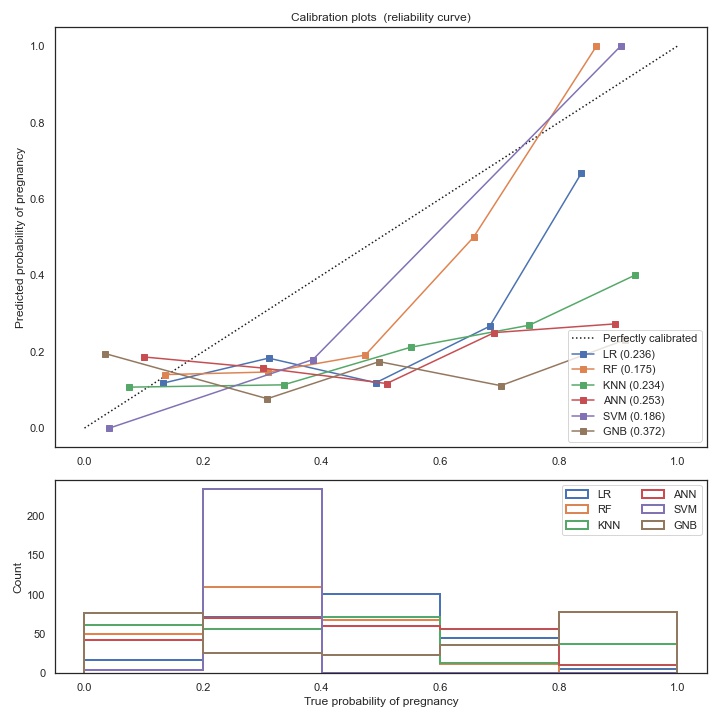


Supplementary Figure 7: Calibration plots (reliability curve) for models in IUI treatment

We show confusion matrix for each of models based on test data with 20% of total sample size (240 of 1197) for IUI treatment in detailed as follows:


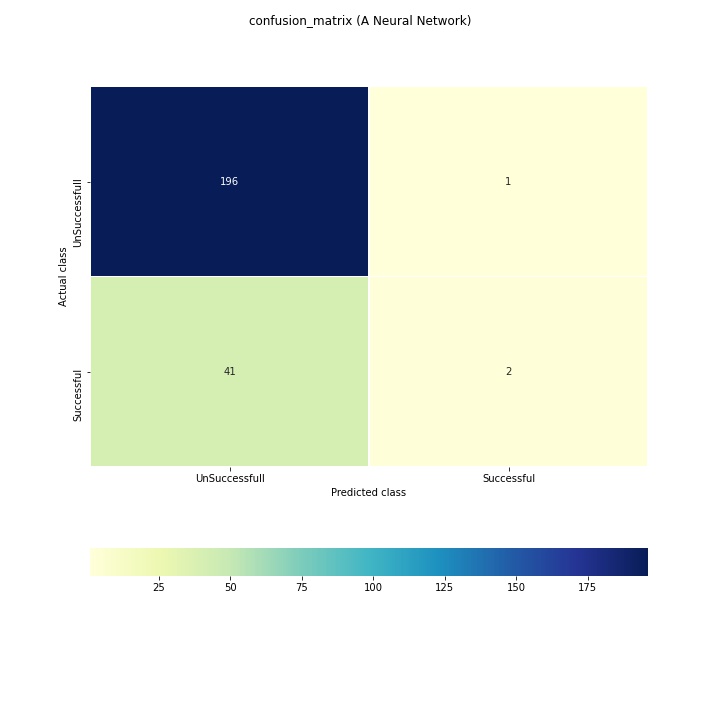

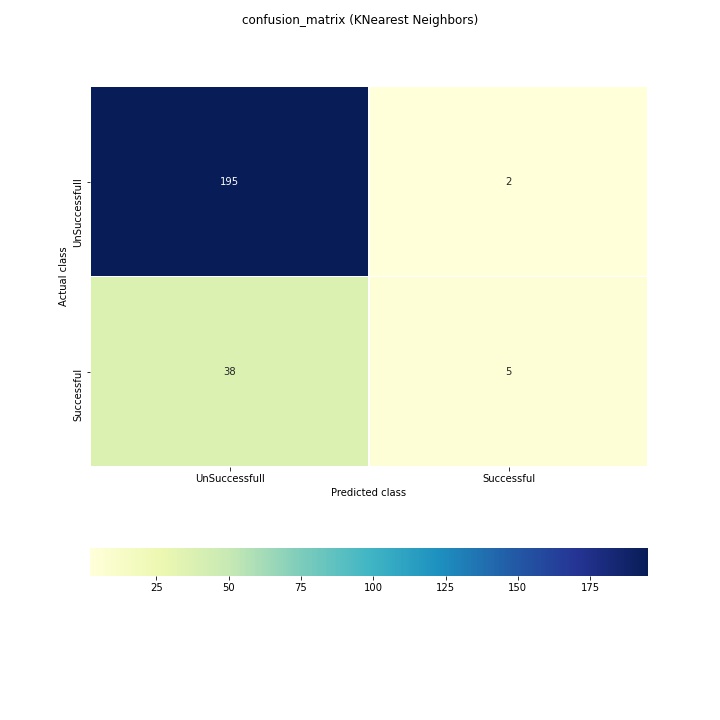

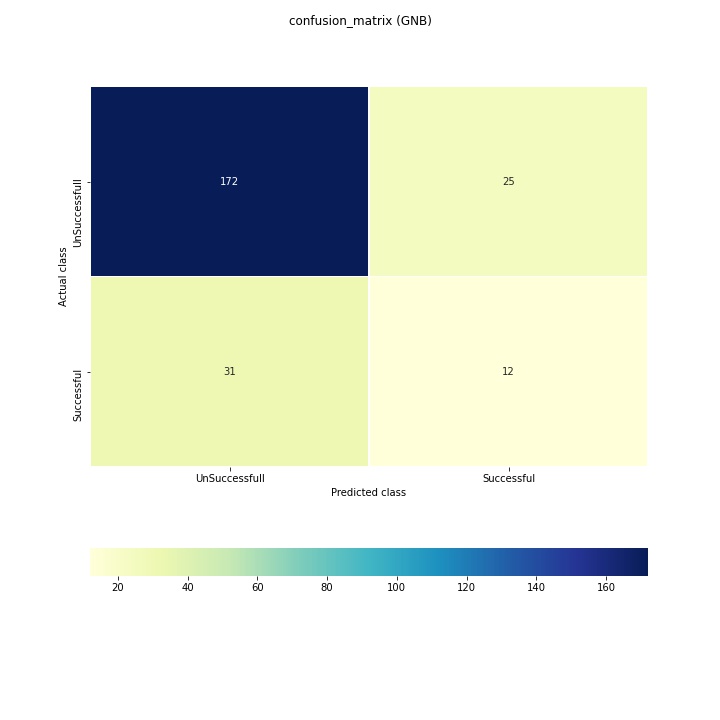

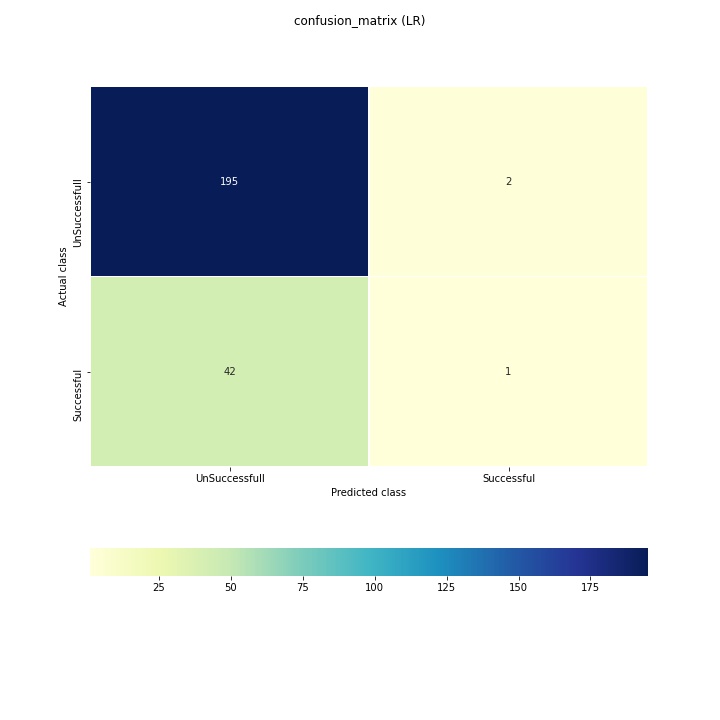

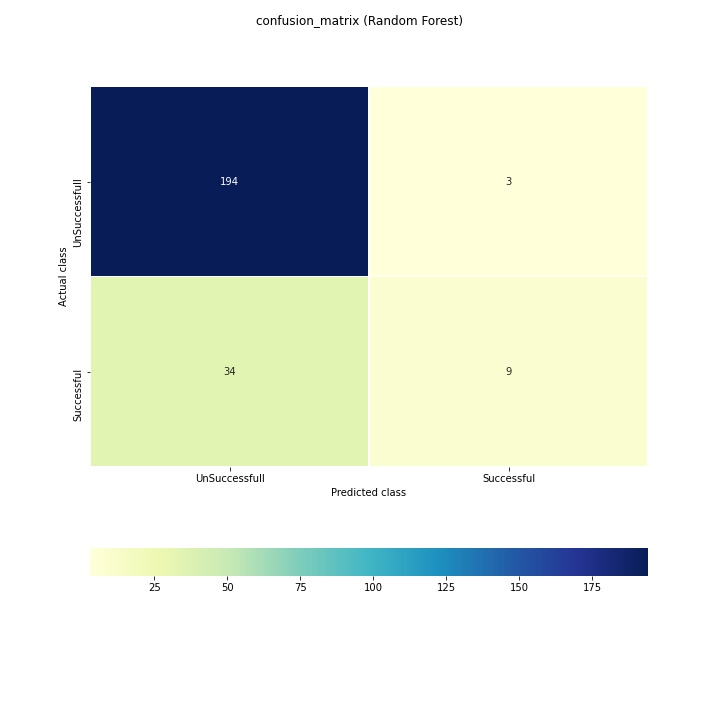

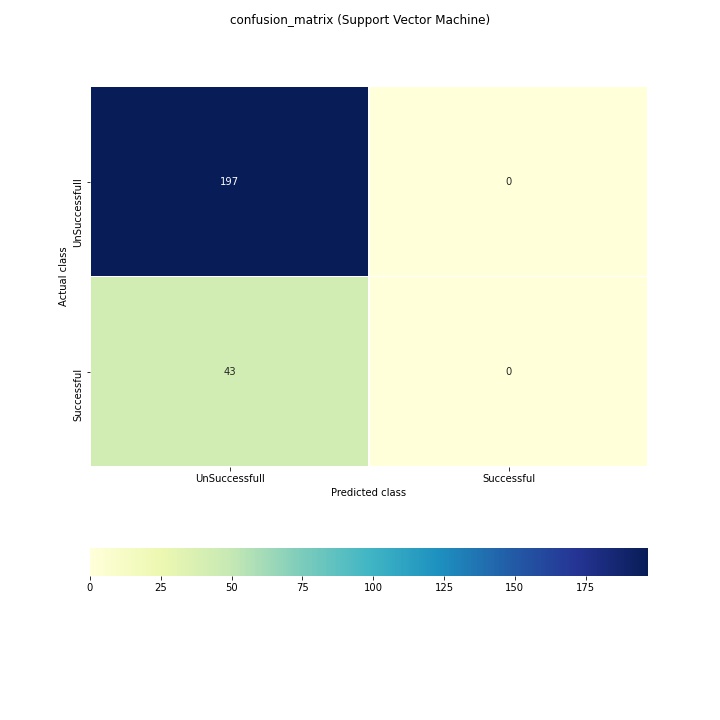


Supplementary Figure 8: Comparison among confusion matrix for models in IUI treatment

Moreover, we used to scikit - learn library (include: with version 0.20 under python version 3.5 platform.

| - Library used for model prediction include: | - Library used for preprocessing data include: |
| --- | --- |
| from sklearn.linear_model import LogisticRegression | from sklearn.model_selection import train_test_split |
| from sklearn.naive_bayes import GaussianNB | from sklearn.model_selection import RandomizedSearchCV |
| from sklearn.neural_network import MLPClassifier | - Libarry used for Analysis results include: |
| from sklearn.neighbors import KNeighborsClassifier | from sklearn.metrics import confusion_matrix |
| from sklearn.ensemble import RandomForestClassifier | from sklearn.metrics import make_scorer, accuracy_score,precision_score,recall_score,f1_score |
| from sklearn import svm | from sklearn.model_selection import cross_val_score |
| - Library used for Visualization include: | from sklearn.metrics import roc_curve |
| import matplotlib.pyplot as plt | from sklearn.metrics import roc_auc_score |
| import seaborn as sns |  |
