# Supplementary material for: Internal validation and comparison of predictive models to determine success rate of infertility treatments: a retrospective study of 2485 cycles
Source: Sci Rep. 2022 May 4;12:7216. doi: 10.1038/s41598-022-10902-9 (PMC9068696; doi:10.1038/s41598-022-10902-9)
Supplement: Supplementary file 2 — Supplementary Information 2. [file 41598_2022_10902_MOESM2_ESM.docx]

**Title: Internal Validation and Comparison of Predictive Models to Determine Success Rate of Infertility Treatments: A Retrospective Study of 2485 Cycles**

**Running Head:** Machine Learning Based Prediction Models in ART Cycles

**Authors:** Ameneh Mehrjerd^a^, Hassan Rezaei^b^, Saeid Eslami^c,d^*, Mariam Begum Ratna^e^, Nayyere Khadem Ghaebi^f^

^a^ Ph. D. Student at Department of Computer Science, Faculty of Mathematics, Statistics and Computer Science, University of Sistan and Baluchestan, Zahedan, Iran.

^b^ Assistant Professor at Department of Computer Science, Faculty of Mathematics, Statistics and Computer Science, University of Sistan and Baluchestan, Zahedan, Iran.

^c^ Department of Medical Informatics, Academic Medical Center, University of Amsterdam, Amsterdam, The Netherlands.

^d^ Associate Professor at Department of Medical Informatics, Faculty of Medicine, Mashhad University of Medical Sciences, Mashhad, Iran

^e^ Research Associate at Department of Epidemiology and Public Health, School of Medicine, University of Nottingham

^f^ Full Professor at Department of Obstetrics & Gynecology, School of Medicine, Mashhad University of Medical Sciences, Mashhad, Iran

***Corresponding Author:** Saeid Eslami, Department of Medical Informatics, Faculty of Medicine, Mashhad University of Medical Sciences, Mashhad, Iran. Tel/Fax: 0098- 5138827048, E-mail: s.eslami.h@gmail.com/ EslamiS@mums.ac.ir

| **Supplementary Table 1**  Clinical characteristics of couples undergoing IVF/ICSI acceptable in infertility centers | | | | |
| --- | --- | --- | --- | --- |
| Characteristic | Successful  (N=240) | Unsuccessful  (N=493) | Total  (N=733) | *p*-value |
| Age  Female  Male | 31.3±5.5  35.4±9.9 | 30.7±5.6  34.8±6.2 | 30.9±5.6  35.3±7.6 | 0.146 ^a^  0.331 ^a^ |
| AFC | 10.1±5.49 | 9.31±5.46 | 9.85±5.49 | 0.059 ^a^ |
| FSH(mIU/ml) | 9.2±4.4 | 6.5±2.4 | 7.4±3.5 | < 0.001 ^a*^ |
| Addiction  Non  Smoke  Alcohol  Narcotic | 139(57.96%)  33(13.75%)  15(6.2%)  53(22.9%) | 269(54.3%)  72(14.4%)  59(11.9%)  93(18.8%) | 407(55.5%)  104(14.18%)  74(10.9%)  148(20.1%) | 0.118 ^c^ |
| Diagnosis  Male Factor  Female Factor  Unexplained  Mix | 100(41.66%)  70(29.1%)  22(9.1%)  48(20%) | 234(47.46%)  118(23.9%)  57(11.5%)  84(17.03%) | 334(45.5%)  188(25.6%)  79(10.7%)  132(18%) | 0.731 ^c^ |
| Duration of Infertility (year) | 6.1±4.7 | 6.0±4.1 | 6.09±4.1 | 0.67 ^a^ |
| Endometrial Thickness(mm) | 9.0±1.8 | 9.4±3.07 | 9.3±2.7 | 0.087 ^a^ |
| Infertility  Primary  Secondary | 185(77.08%)  55(22.9%) | 369(74.8%)  124(25.1%) | 554(75.5%)  179(24.4%) | 0.535 ^b^ |
| Sperm  Count  Motility  Morphology | 54.1±49.8  35.3±22.7  32.3±22.8 | 52.3±59.9  35.6±31.9  31.7±31.4 | 59.2±56.7  35.5±29.2  31.9±28.8 | 0.675 ^a^  0.876 ^a^  0.823^a^ |
| Total Gonadotropin Dose | 32.8±13.7 | 31.0±13.3 | 31.6±13.4 | 0.08 ^a*^ |
| Treatment Cycle Number  Cycle1  Cycle 2  Cycle 3 | 180(75%)  49(20.4%)  11(4.5%) | 350(70.9%)  108(21.9%)  35(7.09%) | 530(72.3%)  157(21.4%)  46(6.2%) | 0.261^c^ |
| No of Follicle | 15.3±22.3 | 19.6±26.4 | 18.2±25.2 | 0.02 ^a*^ |
| BMI | 25.4±4.4 | 25.6±4.0 | 25.5±4.0 | 0.6 ^a^ |
| No of Abs | 0.13±0.4 | 0.17±0.4 | 0.15±0.4 | 0.267 ^a^ |
| No of EP | 0.05±0.2 | 0.05±0.2 | 0.05±0.2 | 0.759 ^a^ |
| No of Frozen Embryos Pack | 1.7±3.5 | 3.1±5.4 | 2.5±4.9 | < 0.001 ^a*^ |
| No of Oocytes Collected | 9.5±13.9 | 11.1±8.2 | 10.5±10.4 | 0.055 ^a^ |
| No of PP LB (IVF/ICSI) | 0.05±0.2 | 0.1±0.3 | 0.08±0.3 | 0.03 ^a*^ |
| No of Pre Unsuccessful IVF/ICSI | 0.1±0.4 | 0.2±0.5 | 0.2±0.5 | 0.123 ^a^ |
| No of Transferred Embryos | 2.3±0.8 | 2.5±0.8 | 2.4±0.8 | < 0.001 ^a*^ |
| No of Viable Oocytes  MI  MII | 0.3±1.5  7.2±5.5 | 0.3±1.2  9.2±6.9 | 0.3±1.3  8.5±6.6 | 0.778 ^a^  < 0.001 ^a*^ |
| Number of cells Day  2Cells  4Cells  6Cells  8 Cells  10 Cells  16 Cells | 0.01±0.11  0.3±0.78  0.06±0.3  1.5±1.06  0.01±0.1  0.07±0.3 | 0.02±0.1  0.3±0.7  0.1±0.4  1.5±1.2  0.02±0.2  0.1±0.5 | 0.015±0.23  0.3±0.7  0.08±0.39  1.5±1.19  0.015±0.1  0.084±0.46 | 0.162 ^a^  0.75 ^a^  0.15 ^a^  0.691 ^a^  0.443 ^a^  0.004 ^a*^ |
| Number of cells Day (Blast) | 0.1±0.5 | 0.2±0.6 | 0.15±0.6 | 0.416 ^a^ |
| Number of cells Day (Compact) | 0.08±0.3 | 0.1±0.4 | 0.09±0.4 | 0.034 ^a*^ |
| Quality of Transferred Embryos  GI  GII  GIII | 1.7±0.9  0.5±0.75  0.004±0.6 | 1.8±0.9  0.7±0.8  0.002±0.2 | 1.78±0.9  0.68±0.8  0.03±0.1 | 0.606 ^a^  0.48 ^a^  0.72 ^a^ |
| freeze/fresh  freeze  fresh | 192(80%)  48(20%) | 376(76.2%)  117(23.7%) | 568(77.4%)  165(22.5%) | 0.149 ^b^ |
| Pre Cycle | 0.42±0.7 | 0.6±0.7 | 0.5±0.7 | 0.092 ^a^ |

Abbreviations: AFC, Antral Follicle Count; FSH, Follicle Stimulating Hormone; BMI, Body Mass Index, Mean ± Standard Division for continues and N (%),

percent of number of couples for categorical variables are presented.

* Significant features (*p*-value <0.05)

^a^ Examined via student’s t-test.
b Examined via Fisher's exact test.
^c^ Examined via Chi-square test

| **Supplementary Table 2**  Clinical characteristics of couples undergoing IUI acceptable in infertility centers | | | | |
| --- | --- | --- | --- | --- |
| Characteristic | Successful  (N=216) | Unsuccessful  (N=980) | Total  (N=1196) | *p*-value |
| Age  Female  Male | 28.8 ± 5.2  32.4±5.0 | 29.4±5.5  33.3±6.3 | 29.3±5.4  33.2±6.1 | 0.147^a^  0.018 ^a*^ |
| AFC | 11.5±5.56 | 10.32±5.14 | 10.5±5.2 | 0.172 ^a^ |
| FSH(mIU/ml) | 6.37±3.3 | 7.3±4.6 | 7.13±4.4 | 0.003 ^a*^ |
| Addiction  Non  Smoke  Alcohol  Narcotic | 202(93.51%)  4(1.85%)  1(0.46%)  7(3.24%)  2(0.92%) | 877(89.48%)  31(3.16%)  1(0.1%)  45(4.59%)  26(2.65%) | 1079(90.2%)  5(2.9%)  2(0.1%)  52(4.3%)  28(2.3%) | < 0.001 ^c*^ |
| Diagnosis  Male Factor  Female Factor  Unexplained  Mix  Ovulatory Dysfunction  Tubal Factor  Diminished Ovarian Reserved | 42(19.44%)  9(4.16%)  53(24.53%)  51(23.67%)  46(21.29%)  6(2.7%)  5(2.3%) | 183(18.6%)  32(3.26%)  274(27.95%)  71(7.2%)  163(16.63%)  81(8.2%)  55(5.61%) | 225(18.81%)  41(3.4%)  326(27.25%)  123(10.2%)  209(17.4%)  87(7.2%)  60(5.01%) | < 0.001 ^c*^ |
| Duration of Infertility (year) | 3.5±2.6 | 4.5±3.2 | 4.3±3.1 | < 0.001 ^a*^ |
| Endometrial Thickness(mm) | 6.9±2.1 | 7.3±2.0 | 7.2±2.0 | 0.27 ^a^ |
| Follicle>16mm | 1.81±1.22 | 1.76±1.3 | 1.78±1.29 | 0.628 ^a^ |
| Infertility  Primary  Secondary | 147(68.05%)  69(31.9%) | 718(73.2%)  262(26.7%) | 865(72.31%)  331(27.67%) | < 0.001 ^b*^ |
| Sperm  Count  Motility  Morphology | 77.92±46.03  50.3±14.5  39.8±21.7 | 85.57±44.4  50.8±14.6  48.9±17.3 | 84.3±45.3  50.7±14.6  47.3±18.4 | 0.021 ^a*^  0.647 ^a^  < 0.001 ^a*^ |
| Total Gonadotropin Dose | 4.99±3.87 | 4.82±3.53 | 4.9±3.54 | 0.04 ^a*^ |
| Treatment Cycle Number  Cycle1  Cycle 2  Cycle 3 | 184(85.18%)  26(12.03%)  6(2.7%) | 786(80.2%)  160(16.32%)  34(3.04%) | 970(81.1%)  186(15.5%)  40(3.34%) | 0.214 ^c^ |
| Use of Drugs  Clomiphene Citrate  HMG  Letrozole  Cinal F  Mix | 7(3.24%)  28(12.96%)  4(38.88%)  0(0.0%)  175(81.01%) | 77(7.85%)  30(3.6%)  24(25.1%)  3(0.3%)  846(86.32%) | 84(7.02%)  58(4.48%)  29(2.12%)  3(0.25%)  1021(85.36%) | < 0.001 ^c*^ |
| BMI | 25.64±3.72 | 25.03±4.03 | 25.12±3.79 | 0.6 ^a^ |

Abbreviations: AFC, Antral Follicle Count; FSH, Follicle Stimulating Hormone; BMI, Body Mass Index, Mean ± Standard Division for continues and N (%),

percent of number of couples for categorical variables are presented.

* Significant features (*p*-value <0.05)

^a^ Examined via student’s t-test.

^b^ Examined via Fisher's exact test.

^c^ Examined via Chi-square test.

| **Supplementary Table 3**  (a) Confusion Matrix | | | | | | | | |
| --- | --- | --- | --- | --- | --- | --- | --- | --- |
|  |  |  | | | Actual | | | |
|  |  | |  | Positive (1) | | |  | Negative (0) |
| Predicted | Positive (1) | |  | TP | |  | FP | |
|  |  | |  |  | |  |  | |
|  | Negative (0) | |  | FN | |  | TN | |
|  | | | | | | | | |
| (b) Evaluation Metric Formulation | | | | | | | | |
| Accuracy $\frac{TP+TN}{N}$ | | | | | | | | |
| Precision $\frac{TP}{TP+FP}$ | | | | | | | | |
| Recall $\frac{TP}{TP+FN}$ | | | | | | | | |
| F-Score $\frac{2*TP}{2*TP+FP+FN}$ | | | | | | | | |

Abbreviations: TP, True Positive; TN, True Negative; FP, False Positive; FN, False Negative; N indicate total samples.
